# The Effects of Text Message and Infographic on Reducing the Number Cigarettes Consumption: A Randomized Controlled Trial

**DOI:** 10.31557/APJCP.2020.21.11.3413

**Published:** 2020-11

**Authors:** Nirun Intarut, Ranee Wongkongdech, Chollada Thronsao

**Affiliations:** 1 *Health Systems Science Unit, Faculty of Medicine, Mahasarakham University, Muang, Maha Sarakham, Thailand. *; 2 *Emergency Medical Operation, Faculty of Medicine, Mahasarakham University, Muang, Maha Sarakham, Thailand. *

**Keywords:** Smoking cessation, text message, randomized controlled trial

## Abstract

**Objective:**

To test the effect of a text-message and infographic to promote smokers quit smoking.

**Methods:**

A randomized control trial was conducted in two provinces of northeast Thailand. Three hundred and ninety-six participants were allocated to either a text-message and infographic group or a control group. We assessed the primary outcome by self-reported 7-day point prevalence smoking abstinence. Multiple logistic regression was used to test the effect of quitting smoking.

**Results:**

At 3-month follow-up, lost to follow-up 16 participants, 380 participants were included for analysis. The difference in the rate of quitting smoking between the intervention and control groups was not found a statistical significance (17.8% versus 11.6%). However, we found a statistically significant difference in the number of cigarettes smokes (the difference: -1.74; 95%CI: -2.63, -0.84).

**Conclusion:**

No effect of text message and infographic for help smokers to quit smoking. However, the intervention showed a decrease in the number of cigarettes smoked.

## Introduction

Estimation of the prevalence of cigarettes smoking showed a decline from 33.3% in 2000 to 20.9% in 2025 (World Health Organization, 2019). This effect might be due to various strategies for tobacco prevention programs, promoting smoking cessation, and enhanced public awareness concerning the harmful effects of smoking and SHS exposure (Levy et al., 2020). As noted, promoting smoking cessation is the one of those strategies to help a smoker quit smoking or to reduce cigarettes consumption. The aims of increasing smoking cessation rate are to reduce morbidity and mortality related to cigarettes smoking. Strategies for smoking cessation involves counselling, quit lines and pharmacotherapy (Hughes, 2003; Shaik et al., 2016).

In Thailand, there have been various strategies used to help smokers to quit smoking, such as 1600 Quitline, or Fah Sai Clinic. Quitline is the main organization responsible for providing smoking cessation consultation (Meeyai et al., 2015). In addition, Fah Sai Clinic was also to help smokers who would like to quit smoking by walking to a primary car unit in each sub-district or a psychological clinic for smoking cessation consultation. With those strategies, report shows the rate of quit smoking at 1, 3, and 6 months follow-up was 49.9%, 38.0% and 33.1%, respectively. Studies also show the effect of pharmacological intervention (Raw et al., 1999), coaching intervention, educational intervention (Reddy et al., 2015; Harvey et al., 2016; Sherman and Smith, 2019), or mHealth (Bock et al., 2013; Haug et al., 2013a) on helping a smoker to quit smoking. Recently, mHealth has been widely employed in health research (Buhi et al., 2013). Reports shown the effectiveness of using mHealth technologies for health promotion, disease prevention, and smoking cessation (Cole-Lewis and Kershaw, 2010; Head et al., 2013; Scott-Sheldon et al., 2016b). In addition, infographics that contains the information of health is easy to understanding and recognizing, and the evidences show the effect of text messaging for motivation a smoker quit smoking (Mussener et al., 2016; Scott-Sheldon et al., 2016a).

Therefore, this study aimed to test the effect of text-messages plus infographic to motivate smokers to quit smoking.

## Materials and Methods

*Research design*

**Figure 1:**
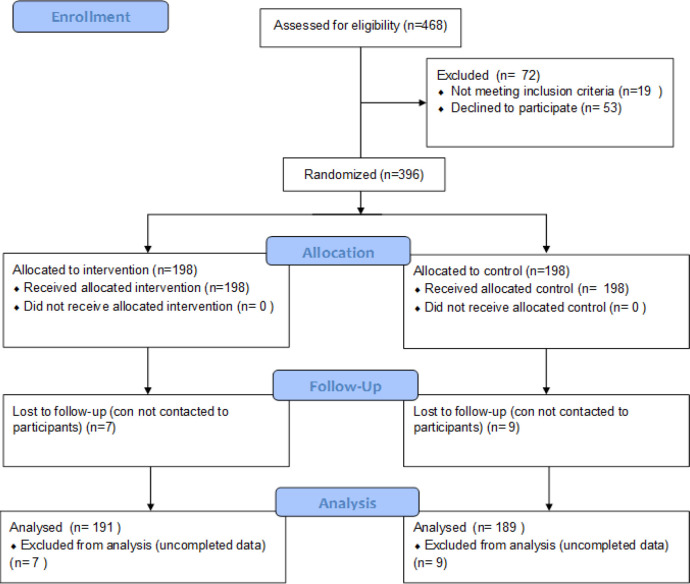
Study Flow Diagram

This study used a randomized controlled trial design and the study flow diagram follows to consort guidelines as shown in Figure 1.

*Participants*

This study was carried out from February 2019 to October 2019 in Maha Sarakham province, and Roi-Et province. For recruitment strategies, we initiated recruiting participants via Facebook advertisements and posters, and then we recruited a participant in the targeted community using the list of smokers in Health Data Center. Inclusion criteria were: (1) a smoker who have a smart phone; (2) a smoker who would like to quit smoking within 1 month. Exclusion criteria was a self-reported having a non-communicable disease because of preventing unexpected events from the disease such as stroke or cardiovascular disease. After screening and collecting the baseline data, we randomized participants to either intervention group or control group. All of the participants in the intervention group received a text-message and infographic for 45 days, automatically and consecutively. The endline assessment was conducted at a 3-month follow-up (Hughes et al., 2003), after the intervention ended.

*Randomization*

We used the computer-base for generating the randomization sequence with block randomization (Kim and Shin, 2014). We used the block of 4 and generated in the website (http://www.randomization.com) (Gerard, 2008). Then the research assistant printed label, managed the randomization schedule, and random allocation to either the intervention or control. The participants were masked regardless of which group they were assigned to.

*Interventions*

This study used a text message for smoking cessation from mCessation. mCessation is a program used to help smokers to quit smoking using mobile technology (mHealth). This program was developed by the Ministry of Health and Family Welfare and the Ministry of Communication and Information Technology in India. A short text concerning message-based mobile health is used to support a smoker to quit smoking (Gopinathan et al., 2018). A professional translator translated the mCessation from English to Thai language. In addition, we created and adapted a picture of an infographic that contained information about the dangers of smoking, tips for quitting smoking, and the benefits of quitting smoking. Therefore, the intervention comprised the text-message plus an infographic picture, which were sent to participants automatically.

Ninety-one of text-messages and 30 infographic pictures were sent to participants. From 1st to 15^th^ day, they received three text-messages and one picture for each day. In total, they received 45 text messages and 15 infographic pictures during 15 days. From 16^th^ to 31^st^ day, the two text messages and one picture were sent to participants daily. During 32^nd^ to 45^th^ day, the participants received one text message and one picture for each day. The example of text-message and infographic presented as the Supplement file 1 and in Supplement file 2. The intervention was delivered to the participants via LINE bot application. LINE is a free application that used for communication. This application also opened for developing for using specific job such as a message notification. We can also write a programing for sending message or picture to targets automatically (Corporation, 2020). Therefore, this study used the Line Messaging API features for developing the LINE bot.

*Outcomes and variables measurement*

*The primary outcome*

Self-reported quit rates. The main outcome was assessed by asking the question “In the past 7 days, have you smoked?” The response options were “yes” or “no”. If participants responded “yes”, they specified the number of cigarettes smoked. Therefore, the definition of quitting smoking was no smoke within the past 7 days.

*Secondary outcomes*

The number of cigarettes consumed in the past 7 days: The number of cigarettes smoked was defined as the number of cigarettes smoked per day in the past 7 days. This outcome was measured from the consecutive question in the primary outcome.

The number of quit attempts. This outcome was defined by asking the question “In the past month, how many times have you tried to quit smoking?” The response option included the number of attempts to quit smoking in the past month.

Quit smoking in the past month. We assessed this outcome by asking the question “In the past month, have you smoked?” The response options were “yes” or “no”. If participants responded “yes”, they specified the number of cigarettes smoked on average in the past month. Therefore, the definition of self-reported quit rates in the past month was not smoking within a previous 30-day period.

*Other variables*

We also collected characteristics comprised of gender, age in years, years attended school, marital status, family income, number of smokers in the family, number of non-smokers in the family, age started smoking, self-confidence in quitting smoking score, and Fagerstrom test for nicotine dependence (FTND).

*Sample size determination*

The sample size was determined by comparing self-reported quit rates between intervention and control group as the following parameters: an alpha error probability of .05, power of 0.80, an effect size of 15%, and adjusted for loss to follow up of 20%. The effect size of self-reported quit rates was estimated base on previous studies (Mussener et al., 2016). This study required a total sample size of 250 participants (125 participants per group).

*Statistical methods*

Descriptive statistics were used to characterize the study sample in the intervention and control groups. We also tested the difference in baseline characteristics between the intervention and control groups by using Chi-square test.

For the primary outcome, we used multiple logistic regression adjusted for baseline characteristics. In addition, the secondary outcomes were assessed by using multiple linear regressions adjusted for the baseline characteristics. We also did subgroups analysis stratified by the place of recruitment that are community and university areas. All analyses were conducted using R software version 3.6.3 (R Core Team, 2019) and epiDisplay package (Chongsuvivatwong, 2018). P < 0.05 was considered statistically significant.

*Ethical approval and informed consent*

This study was approved by the Mahasarakham university Institutional Review Board (IRB) approved the study with identification number 113/2561. The written consent forms were distributed, provided and signed to all participants

*Clinical Trial Registration*

This study has been registered at the Thai Clinical Trials Registry (TCTR) with study identification as TCTR20190213004.

## Results

Figure 2 shows the study flow from assessed eligibility to analyzed data. Four hundred and sixty-eight participants were asked to participate in this study. Of those, 84.6% (n = 396) were included and randomized to either the intervention (n = 198) or control (n = 198) groups. After follow-up at 3 months, we assessed endline with 191 participants (Lost to follow-up: 7) in the intervention group and with 189 participants (Lost to follow-up: 9) in the control group.

*Baseline characteristics*

Baseline characteristics are presented in Table 1. Males comprised 98.9%, while 39.1% were aged 26-35 years. 80% attended school for more than 6 years. Approximately 69.7% participants were students, merchants, employed, or freelance. Overall, 41.1% of participants reported being addicted the nicotine at a low level. Comparing the baseline characteristics, we observed the non-statistical significance comprised as gender, years attended school, occupation, marital status, family income, and number of non-smokers in the family, as well as Fagerstrom Test for Nicotine Dependence and self-confidence to quit smoking score. However, we observed statistical significance between the intervention and control groups in terms of participants’ ages, number of smokers in the family, and duration of smoking.

*Outcomes assessment*

Table 2 shows the univariate analysis of primary outcome and secondary outcomes. For the primary outcome, 17% of participants had quit smoking in the intervention groups, while 11.6% in the control group had quit with non-statistical significance. In addition, there was no difference in the rate of quitting smoking within 30 days between the intervention and control groups. For the endline assessment, there were statistical significance differences in the number of cigarettes smoked in the past 30 days (p value: < 0.001) and in the past 7 days (p value: < 0.001).

The multivariable analysis results are shown in Table 3. There was a non-statistical significance in 7-day point prevalence quitting smoking rate (Adjusted OR: 1.74; 95%CI: 0.93, 3.23) and 30-day prolonged abstinence (quit smoking rate) (Adjusted OR: 1.02; 95%CI: 0.49, 2.12). Multiple linear regression shows a number of cigarettes smoked in the past 30 days for the intervention was lower than for the control group (difference: -1.74; 95%CI: -2.63, -0.84) with statistical significance. However, the number of quit attempts (difference: -0.15; 95%CI: -0.39, 0.09) and the number of cigarettes smoked in the past 7 days (difference: 0.53; 95%CI: -0.34, 1.41) in the intervention group was not statistical significance difference between the intervention and control group. In addition, the result of subgroup analysis, 7-day point prevalence quitting smoking rate, shown that there had not differences in odds of quitting smoking between community and university (MH p-value: 0.3943).

## Discussion

This study shows evidence of the effect of text-messages for encouraging smokers try to reduce the number of cigarettes smoked in the past 30 days. However, we could not observe statistical significance in quitting smoking in the past 7 days or 30 days, the number of cigarettes smoked in the past 7 days, or the number of quit attempts in the past 30 days.

Comparing our results to other studies, our primary finding shows non-significance in the difference for quitting smoking rate. This finding was different from some studies (Skov-Ettrup et al., 2014; Gopinathan et al., 2018; Liao et al., 2018; Gram et al., 2019) and similar to other studies (Ybarra et al., 2012; Haug et al., 2013b; Naughton et al., 2014; Stanczyk et al., 2014; Skov-Ettrup et al., 2016). Most significance studies tested participants with health condition problems and in clinic settings. This study was based on a community setting where participants were healthy. However, evidence reported successful smoking cessation intervention in unhealthy volunteers (Samaan et al., 2012; Pires-Yfantouda et al., 2013; Guilleminault et al., 2018) and observational study shows the association between unhealthy status and quit smoking (Li et al., 2019). We observed the significance of the number of cigarettes smoked per day. Similar findings showed there was a decrease in the number of cigarettes smoked per day on average (Haug et al., 2013b; Liao et al., 2018). A smoker who would like to quit smoking is suggested to first reduce cigarette smoking then they can attempt to stop smoking in the future (Lindson et al., 2019). Additional evidence shows reducing cigarette smoking has the potential for increasing quit attempts and quitting smoking in the future (Cropsey et al., 2011).

This study had several limitations. First, we recruited participants from several places such as university students staying near dormitories around the university, as well as those in the community. During the recruitment period, it was difficult to invite participants. For recruitment of university students, we employed students part-time to paste brochures around the dormitories and invite their friends to participate in the study. For the community, we invited participants through health volunteer workers, gaining a higher rate of participation. This difference in the research team and place of recruitment might affect to our findings. However, we did the subgroup analysis and the results shown that there had not difference odds of the quitting smoking rate. Second, our study could not verify the quit-smoking status by biochemical verification. Thus, the results might be distorted because of information bias (Cunningham and Kushnir, 2013; Pierce et al., 2019). Finally, our randomization could not be completed because there was statistical significance in some variables. However, we used a statistical model that included all baseline characteristics (Roberts and Torgerson, 1999). For the strength of the study, this study might be the first study to test the effect of text-message plus infographic on smoking cessation in Thailand. In addition, the benefits of this intervention include low cost, less staff, the messages sent to participants by personality, and automatic message sending.

In conclusion, our finding could not find evidence for quitting smoking. However, the intervention shows a decrease in the number of cigarettes per day. These findings should be tested further in clinical settings, including biochemical testing and the significance of long-term effects.

**Table 1:** Baseline Characteristics and Univariable Analysis of Participants by Intervention Groups

Variables	Total N=380	Control N=189	Intervention N=191
Gender			
Male	376 (98.9)	188 (99.5)	188 (98.4)
Female	4 (1.1)	1 (0.5)	3 (1.6)
Age (years) ^A^			
18-25y	82 (22.1)	38 (20.7)	44 (23.5)
26-35y	145 (39.1)	60 (32.6)	85 (45.5)
36y	144 (38.8)	86 (46.7)	58 (31)
Duration of school attendance (years)			
≤ 6	76 (20)	38 (20.1)	38 (19.9)
> 6	304 (80)	151 (79.9)	153 (80.1)
Occupation			
Agriculture	40 (10.5)	19 (10.1)	21 (11)
Others (student, merchant, employed, freelance)	265 (69.7)	136 (72)	129 (67.5)
Civil service	43 (11.3)	18 (9.5)	25 (13.1)
Unemployed	32 (8.4)	16 (8.5)	16 (8.4)
Marital status			
Married	121 (31.8)	54 (28.6)	67 (35.1)
Single/Divorce/Separated	259 (68.2)	135 (71.4)	124 (64.9)
Family income per month (Thai baht)			
<10,000	247 (65)	131 (69.3)	116 (60.7)
≥10,000	133 (35)	58 (30.7)	75 (39.3)
Number of smokers in house (persons) ^A^			
1	237 (62.4)	104 (55)	133 (69.6)
≥ 2	143 (37.6)	85 (45)	58 (30.4)
Number of non-smokers in house (persons)			
< 4	319 (83.9)	158 (83.6)	161 (84.3)
≥ 4	61 (16.1)	31 (16.4)	30 (15.7)
Duration of tobacco smoking (years) ^A^			
1-10	113 (29.7)	48 (25.4)	65 (34)
11-20	144 (37.9)	65 (34.4)	79 (41.4)
≥ 21	123 (32.4)	76 (40.2)	47 (24.6)
Fagerstrom Test for Nicotine Dependence			
Low	156 (41.1)	70 (37)	86 (45)
Moderate	144 (37.9)	76 (40.2)	68 (35.6)
High	80 (21.1)	43 (22.8)	37 (19.4)
Self-confidence in quit smoking score			
Mean (SD)	4 (2.6)	4 (2.6)	4 (2.6)

A, mean there was a statistical significance

**Table 2:** The Result of Univariable Analysis of Primary and Secondary Outcomes

Primary Outcome	Pre testing		P value	Post testing		P value
7-day quit smoking n (%) Secondary Outcomes	-	-		34 (17.8)	22 (11.6)	0.09 ^A^
30-day quit smoking n (%)	-	-		19 (9.9)	19 (10.1)	0.973 ^A^
The number of cigarettes consumed in the past 30 days mean (sd)	6.6 (3.3)	6.3 (2.5)	0.257 ^B^	5.3 (2.6)	6.6 (3.2)	< 0.001 ^B^
The number of cigarettes consumed in the past 7 days mean (sd)	5.6 (2.3)	8.1 (3.1)	< 0.001 ^B^	4.1 (2.3)	6.2 (3)	< 0.001 ^B^
The number of quit attempts in the past 30days mean (sd)	0.6 (1)	0.4 (0.9)	0.018 ^B^	0.4 (0.7)	0.2 (0.4)	0.007 ^B^

^A^, performed by using Chi-square test; ^B^, performed by using unpaired t-test

**Table 3:** The Result of Multivariable Aanalysis of Primary and Secondary Outcomes Assessment

Outcomes	All	Community	University
Primary outcome	Adjusted OR (95%CI)		
7-day quit smoking ^A^	1.74 (0.93,3.23)	1.53 (0.77,3.06)	3.33 (0.76,14.7)
Secondary outcomes			
30-day quit smoking ^A^	1.02 (0.49, 2.12)	0.92 (0.4,2.09)	1.78 (0.37,8.62)
The number of cigarettes consumed in the past 30 days ^B^	-1.74 (-2.63, -0.84)	-1.82 (-2.82, -0.83)	-1.84 (-3.63, -0.05)
The number of cigarettes consumed in the past 7 days ^B^	0.53 (-0.34, 1.41)	0.76 (-0.17, 1.69)	-1.77 (-4.22, 0.69)
The number of quit attempts in the past 30 days ^B^	-0.15 (-0.39, 0.09)	-0.21 (-0.47, 0.05)	0.07 (-0.45, 0.58)

All and community, Model A: Multiple logistic regression model adjusted for gender, age, duration of school attendance, occupation, marital status, family income per month, number of smokers in house, number of non-smokers in house, duration of tobacco smoking, fagerstrom test for nicotine dependence, and self-confidence in quit smoking score; Model B: Multiple linear regression model adjusted for baseline, gender, age, duration of school attendance, occupation, marital status, family income per month, number of smokers in house, number of non-smokers in house, duration of tobacco smoking, fagerstrom test for nicotine dependence, and self-confidence in quit smoking score; University, Model A: Multiple logistic regression model adjusted gender, family income, number of smokers in house, number of non-smokers in house, fagerstrom test for nicotine dependence, and self-confidence in quit smoking score; Model B: Multiple linear regression model adjusted for baseline, gender, family income, number of smokers in house, number of non-smokers in house, fagerstrom test for nicotine dependence, and self-confidence in quit smoking score.
